# Augmented Reality for Perioperative Anxiety in Patients Undergoing Surgery: A Randomized Clinical Trial

**DOI:** 10.1001/jamanetworkopen.2023.29310

**Published:** 2023-08-17

**Authors:** Michael G. Rizzo, Joseph P. Costello, Dylan Luxenburg, Jacob L. Cohen, Nicolas Alberti, Lee D. Kaplan

**Affiliations:** 1 UHealth Sports Medicine Institute, Department of Orthopaedics, University of Miami, Miami, Florida; 2 Miller School of Medicine, University of Miami, Miami, Florida; 3 Center for Computational Science, University of Miami, Miami, Florida

## Abstract

**Importance:**

Both augmented reality (AR) and virtual reality (VR) have had increasing applications in medicine, including medical training, psychology, physical medicine, rehabilitation, and surgical specialties, such as neurosurgery and orthopedic surgery. There are little data on AR’s effect on patients’ anxiety and experiences.

**Objective:**

To determine whether the use of an AR walkthrough effects patient perioperative anxiety.

**Design, Setting, and Participants:**

This randomized clinical trial was conducted at an outpatient surgery center in 2021 to 2022. All patients undergoing elective orthopedic surgery with the senior author were randomized to the treatment or control group. Analyses were conducted per protocol. Data analysis was performed in November 2022.

**Intervention:**

AR experience explaining to patients what to expect on their day of surgery and walking them through the surgery space. The control group received the standard educational packet.

**Main Outcomes and Measures:**

The main outcome was change in State-Trait Anxiety Inventory (STAI) from the screening survey to the preoperative survey.

**Results:**

A total of 140 patients were eligible, and 45 patients either declined or were excluded. Therefore, 95 patients (63 [66.3%] male; mean [SD] age, 38 [16] years) were recruited for the study and included in the final analysis; 46 patients received the AR intervention, and 49 patients received standard instructions. The AR group experienced a decrease in anxiety from the screening to preoperative survey (mean score change, −2.4 [95% CI, −4.6 to −0.3]), while the standard care group experienced an increase (mean score change, 2.6 [95% CI, 0.2 to 4.9]; *P* = .01). All patients postoperatively experienced a mean decrease in anxiety score compared with both the screening survey (mean change: AR, −5.4 [95% CI, −7.9 to −2.9]; standard care, −6.9 [95% CI, −11.5 to −2.2]; *P* = .32) and preoperative survey (mean change: AR, −8.0 [95% CI, −10.3 to −5.7]; standard care, −4.2 [95% CI, −8.6 to 0.2]; *P* = .19). Of 42 patients in the AR group who completed the postoperative follow-up survey, 30 (71.4%) agreed or strongly agreed that they enjoyed the experience, 29 (69.0%) agreed or strongly agreed that they would recommend the experience, and 28 (66.7%) agreed or strongly agreed that they would use the experience again. No differences were observed in postoperative pain levels or narcotic use.

**Conclusions and Relevance:**

In this randomized clinical trial, the use of AR decreased preoperative anxiety compared with traditional perioperative education and handouts, but there was no significant effect on postoperative anxiety, pain levels, or narcotic use. These findings suggest that AR may serve as an effective means of decreasing preoperative patient anxiety.

**Trial Registration:**

ClinicalTrials.gov Identifier: NCT04727697

## Key Points

**Question:**

Does a preoperative walkthrough of a patient’s day of surgery using augmented reality (AR) effect the patient’s anxiety levels?

**Findings:**

In this randomized clinical trial that included 95 patients, patients who received the preoperative AR experienced a significant decrease in preoperative State-Trait Anxiety Inventory (STAI) score compared with the control group that received standard education materials. There was no significant difference in STAI scores after operations.

**Meaning:**

These findings suggest that a preoperative AR walkthrough may be a useful tool for reducing preoperative anxiety, but its impact on postoperative anxiety is less clear.

## Teaser

This randomized clinical trial assesses whether the use of an augmented reality walkthrough of the day of surgery reduces patient perioperative anxiety compared with a standard educational control.

## Introduction

Anxiety experienced by patients before a surgical intervention is a well-documented phenomenon, occurring in up to 60% to 80% of patients, and it can lead to alterations in cognitive and physiologic function.^1,2,3^ Autonomic dysfunction occurs as a result of undermanaged anxiety, resulting in biochemical imbalances shown to cause increased perioperative pain, anesthesia requirements, and analgesic medication requirements.^4,5^ These adverse effects can ultimately lead to longer hospital stays, increased duration of recovery—both physical and behavioral—and ultimately diminished satisfaction.^4,6,7^ Several studies have identified independent risk factors in patients predisposed to a preoperative state of anxiety, including high baseline anxiety level, psychiatric disorder, negative perception, the extent of the proposed surgery, having a higher American Society of Anesthesiologists (ASA) grading, history of smoking, education level, female gender, and previous adverse clinical experience.^8,9^

There is strong evidence that preoperative educational interventions can reduce patient perioperative anxiety.^7,9,10,11,12^ These interventions often aim to improve patients’ knowledge about the presurgical and postsurgical events, the operation they will undergo, and their postoperative management and rehabilitation.^5,11^ These are typically performed by a nurse or care team members and typically involve individual sessions, handouts, visual aids, or videos.^10^

Advances in the preparation and delivery of patient care and education have begun to incorporate augmented reality (AR) and virtual reality (VR). AR has been defined as the superimposition of interactable virtual objects onto a physical environment in space.^13^ Alternatively, VR substitutes a user’s complete visual environment with a computer-generated landscape. These technologies have gained much interest with applications in gaming, home layout, and navigation.^14^ Within medicine, the use of AR and VR is still in its infancy, but they have begun to be used in a multitude of settings, including both medical training and patient care.^15^ Limited reviews within the literature have suggested that AR and VR are successful tools for patient education and satisfaction.^16,17^

AR and VR have shown therapeutic benefits in psychology,^18^ physical medicine, and rehabilitation^19^ and more broadly as a tool for pain and anxiety reduction when used as a distraction to decrease the perception of reality during hospitalization, venipuncture, wound care, chemotherapy, physical therapy, and routine dental and endoscopic procedures.^20,21,22,23,24,25,26^ This has even been demonstrated to be an effective adjunct in surgical procedures using wide-awake local anesthesia, regional anesthesia, and spinal anesthesia.^27,28,29,30,31^

While AR and VR have been used to improve patient education about the specifics of their procedure, to our knowledge, no studies have been performed that examine AR’s ability to affect patient anxiety and experience in the outpatient surgical setting by providing the patient a step-by-step walkthrough of their day of surgery. We hypothesize that by applying the AR to perioperative patient education and experience, we can decrease perioperative patient anxiety and improve patient experience and satisfaction.

## Methods

### Trial Design

This randomized clinical trial was approved by the institutional review board at the University of Miami. All participants provided verbal informed consent or assent. This study followed the Consolidated Standards of Reporting Trials (CONSORT) reporting guideline. The trial protocol and statistical analysis plan are presented in Supplement 1.

Participants were recruited from the University of Miami and included patients who were indicated to have an elective, outpatient orthopedic surgery with the senior author (L.D.K.). Recruitment began in November 2021, and the follow-up for the final patient was completed in October 2022.

**Figure 1.**
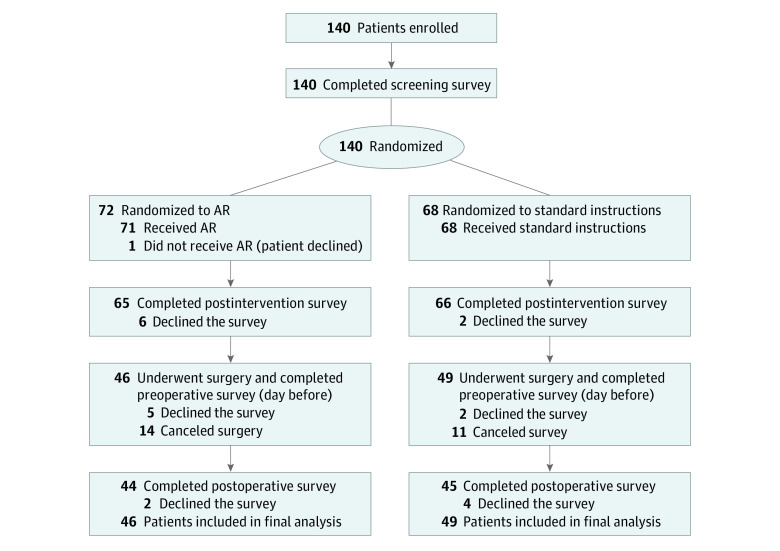
Trial Flowchart and Survey Schedule

To investigate the effect of a preoperative AR training experience on patient anxiety, we randomized 140 patients to receive either the AR experience or the standard set of preoperative instructions as a control. The interactive AR experience was a custom application developed in-house in collaboration with our institution’s Center for Computational Science by Xennial Digital Studios. The AR intervention visually walks patients through their trip to the operating room with narration from their surgeon while using an AR headset. The development was funded by an institutional grant of $10 000 that covered the costs of the application design and development. The AR experience takes approximately 3 minutes. Inclusion criteria consisted of all patients aged 12 years or older who were scheduled to have an ambulatory procedure with our senior author. Patients were excluded if they did not speak English (as the AR application was recorded only in English) or if they had circumstances that would prevent them from meeting or performing study requirements, such as canceling surgery or not completing the required surveys. After patients were indicated for surgery and given a surgical date, verbal informed consent was obtained. Patients were then randomized into 1 of 2 groups (AR or control) using a randomization table by whoever was performing the enrollment (M.G.R, J.P.C., or D.L.) (Supplement 2). Patients assigned to the control group received only the standard surgical instructions packet provided to all patients at our center. Patients in the AR group received both the standard surgical instruction packet and the AR experience. The participant recruitment flowchart is depicted in Figure 1.

### Outcomes

To quantify anxiety levels, we used the State-Trait Anxiety Inventory (STAI; range, 20-80; higher score indicates a higher level of anxiety), which is a validated and widely used survey to determine patients’ anxiety.^32^ It was administered at 4 different time points to both the control and AR groups (screening, before the intervention, before the operation, and after the operation) to quantify levels of patient anxiety. The STAI was administered at all points and for all groups via an online survey system. The screening STAI was administered immediately after enrollment to the study for both groups and served as a baseline anxiety index for each patient. After completing the preintervention STAI, patients in the AR group used the AR headset to receive preoperative educational training. Patients assigned to the control group did not receive any type of additional verbal, written, video, AR, or other source of education. On completion of the AR preoperative education intervention for the AR group and after a brief waiting period for the control group (3-5 minutes), the postintervention STAI was administered to measure any immediate effect of the preoperative education between the groups. This concluded their preoperative visit. The night before their scheduled procedures, patients in both groups were contacted and asked to fill out the presurgery STAI to assess anxiety at that time. Following their surgical procedure, patients in both groups were contacted at the time of their first postoperative visit to complete the postoperative STAI survey. Our primary end point was the difference in STAI from screening survey to preoperative survey, with secondary end points screening to postintervention survey, screening to postoperative survey, and preoperative to postoperative survey. We additionally collected patient-reported pain scores based on the visual analog scale (VAS; range, 0-10; higher score indicates a greater level of pain) and the number of pain pills used on postoperative day 1 and at the time of their postoperative visit 7 to 10 days after surgery, in addition to overall feedback and satisfaction with the experience.^33,34^

### Statistical Analysis

An a priori power analysis was conducted to determine the number of patients needed to detect a difference in STAI scores between the groups. To detect a clinically meaningful difference of a change of 5 points in the STAI with a power of 0.8, a significance level of 0.05, and an SD of 8.9, 51 patients were needed in each group. The SD used was based on a separate study involving patients undergoing procedures under general anesthesia.^10^

To determine whether we were appropriately powered to detect a clinically meaningful difference of 2 points in VAS, we conducted a post-hoc power analysis: with 80% power to detect α = .05, based on our VAS SD of 3.12, we required 40 patients in each group; as there were only 24 patients who completed the pain portion of the postoperative survey, we did not reach appropriate confidence for definitive conclusion for this comparison.

Continuous variables are presented as mean and SD. Categorical variables are presented as count and percentage. Mann-Whitney *U* tests were used to compare continuous data, and Fisher Exact tests were used to compare categorical data, unless otherwise indicated. Significance was assessed at *P* < .05, and all *P* values were 2-tailed. All data analysis was performed using Python version 3.8.10 (Python Software Foundation) and R version 4.2.1 (R Project for Statistical Computing) software, with the libraries RPy2 3.5.4, NumPy 1.23.3, and SciPy 1.9.1 for Python and Stats 4.2.1 for R. Data analysis was performed in November 2022.

## Results

Of 140 eligible patients, 45 patients either declined or were excluded; therefore, 95 patients (63 [66.3%] male; mean [SD] age, 38 [16] years; range, 14-78 years) were recruited for the study and included in the final analysis, with 46 patients randomized to receive the AR intervention and 49 patients randomized to the standard instructions control group. There were no significant differences between AR and control groups with respect to age (mean [SD], 38 [16] years vs 38 [17] years), sex (30 [65.2%] male vs 33 [67.3%] male), ASA score (eg, ASA class 1: 23 patients [50.0%] vs 23 patients [46.9%]), smoking history (eg, never smoker: 40 patients vs 41 patients), psychiatric comorbidity (1 patient [2.2%] vs 6 patients [12.2%]), surgical location (eg, knee: 33 patients [71.7%] vs 31 patients [63.3%]), days between the preoperative appointment and date of surgery (mean [SD], 28 [32] days vs 26 [23] days), or screening STAI score (mean [SD], 35 [10] vs 35 [12]) (Table 1). A total of 95 patients underwent the appropriate intervention and completed the necessary preoperative surveys: 46 patients received the AR intervention, and 49 patients received the standard instructions. A total of 6 patients completed all necessary steps and surveys before surgery, but ultimately declined the final postoperative survey: 2 patients in the AR intervention group and 4 patients in the standard instructions group.

**Table 1.** Basic Demographics and Clinical Factors Between Groups

Characteristic	AR (n = 46)	Control (n = 49)
Age (SD), y	38 (16)	38 (17)
Sex		
Male	30 (65.2)	33 (67.3)
Female	16 (34.8)	16 (32.7)
ASA Score^a^		
1	23 (50.0)	23 (46.9)
2	21 (45.7)	24 (49)
3	1 (2.2)	2 (4.1)
Smoking history		
Never	40 (87.0)	41 (83.7)
Former	4 (8.7)	5 (10.2)
Current	2 (4.3)	3 (6.1)
Psychiatric comorbidity		
Yes	1 (2.2)	6 (12.2)
No	45 (97.8)	43 (87.8)
Surgery location		
Knee	33 (71.7)	31 (63.3)
Shoulder	9 (19.6)	16 (32.7)
Elbow	3 (6.5)	2 (4.1)
Time from preoperative visit to surgery (SD), d	28 (32)	26 (23)
Screening STAI (SD) score^b^	35 (9.8)	35 (12)

Abbreviations: AR, augmented reality; ASA, American Society of Anesthesiologists; STAI, State-Trait Anxiety Inventory.

^a^ ASA score was not available for 1 patient in the AR group. There were no patients with ASA class 4 or higher included in the study.

^b^ Range, 20 to 80; higher scores indicate greater levels of anxiety.

To determine whether patients who received the AR experience had a change in their preoperative anxiety, we compared the differences in postintervention and preoperative STAI scores vs screening scores (Table 2). There was a significant mean difference between the AR group, which experienced a decrease of 3.1 (SD, 5.7; 95% CI, −5.0 to −1.1) points from the screening survey to the postintervention survey compared with the standard care group, which had no change (mean difference, 0.1 [SD, 3.7; 95% CI, −1.2 to 1.3] points; *P* = .01). There also was a significant mean difference between the groups regarding screening to preoperative surveys, with the AR group again demonstrating a decrease in STAI (mean difference, −2.4 [SD, 7.3; 95% CI, −4.6 to −0.3] points) while the standard group showed an increase (mean difference, 2.6 [SD, 8.4; 95% CI, 0.2 to 4.9] points; *P* = .01). These data suggest that the AR intervention decreased preoperative anxiety not only immediately—as evidenced by the initial decrease in STAI—but also with a lasting effect until the time of surgery.

**Table 2.** Changes in State-Trait Anxiety Inventory Scores Between Groups at Different Survey Points

Comparison	AR		Control		*P* value
	No.	Score difference, mean (SD) [95% CI]	No.	Score difference, mean (SD) [95% CI]	
Screening vs postintervention survey	36	−3.1 (5.7) [−5.0 to −1.1]	37	0.1 (3.7) [−1.2 to 1.3]	.01
Screening vs preoperative survey	46	−2.4 (7.3) [−4.6 to −0.3]	49	2.6 (8.4) [0.2 to 4.9]	.01
Screening vs postoperative survey	44	−5.4 (8.3) [−7.9 to −2.9]	45	−6.9 (15.6) [−11.5 to −2.2]	.27
Preoperative vs postoperative survey	44	−8.0 (7.6) [−10.3 to −5.7]	45	−4.2 (14.8) [−8.6 to 0.2]	.19

Abbreviation: AR, augmented reality.

We additionally compared the changes from the screening and preoperative surveys to postoperative surveys (Table 2). There was no significant mean difference in STAI between groups from either screening to postoperative surveys (Table 2). We also performed subgroup analysis using analysis of variance between the patients who received the AR intervention based on the anatomic location of surgery. There were no differences between change in STAI in any of the end points between anatomic location (Table 3).

**Table 3.** Changes in State-Trait Anxiety Inventory Scores for the Augmented Reality Group Between Survey Points by Body Part^a^

Comparison	Knee		Shoulder		Elbow		*P* value
	No.	Mean (SD) [95% CI]	No.	Mean (SD) [95% CI]	No.	Mean (SD) [95% CI]	
Screening vs postintervention survey	28	−3.7 (6.0) [−6.0 to −1.4]	5	1.0 (3.1) [−2.8 to 45.8]	2	−4.0 (5.7) [−54.8 to 46.8]	.24
Screening vs preoperative survey	33	−3.0 (7.8) [−5.8 to −0.3]	9	−0.22 (6.6) [−5.3 to 4.8]	3	−2.0 (2.6) [−8.6 to 4.6]	.60
Screening vs postoperative survey	31	−5.7 (9.2) [−9.1 to −2.4]	9	−6.3 (5.8) [−10.8 to −1.9]	3	−1.7 (6.0) [−16.6 to 13.3]	.70
Preoperative vs postoperative survey	31	−9 (7.9) [−11.9 to −6.1]	9	−6.6 (7.4) [−12.2 to −0.9]	3	−3.7 (4.2) [−14.0 to 6.7]	.41

^a^ Analyses were performed using analysis of variance. .

To determine whether the AR intervention influenced postoperative pain or narcotic use, patients were asked their pain levels as well as narcotic use both on postoperative day 1 and at the time of the postoperative appointment (7 to 10 days after surgery). There were no significant differences between groups with respect to postoperative pain or narcotic pill use on postoperative day 1 or at the time of the postoperative appointment (eTable in Supplement 2).

**Figure 2.**
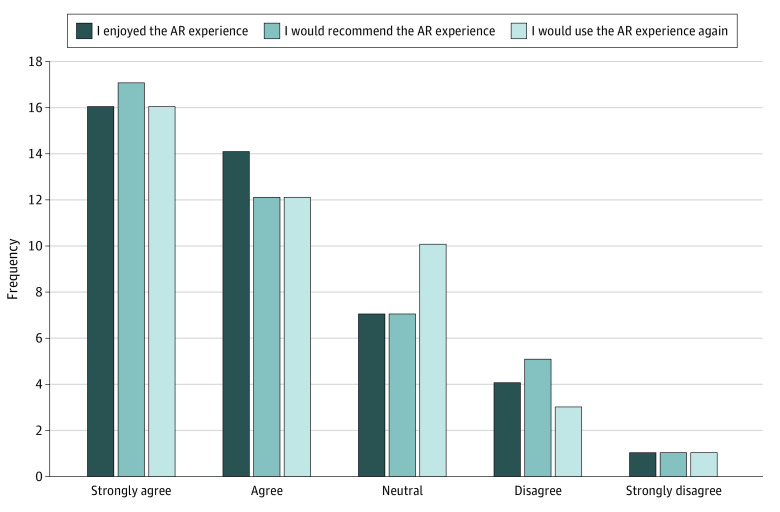
Augmented Reality (AR) Group Experiences With the AR Application

Among patients who underwent the AR intervention, 42 patients were additionally asked about their overall experience with the AR application. Most patients either agreed or strongly agreed that they enjoyed the AR experience (30 patients [71.4%]), would recommend the experience (29 patients [69.0%]), and would do it again (28 patients [66.7%]) (Figure 2). However, some patients disagreed or strongly disagreed that they enjoyed the experience (5 patients [11.9%]), would recommend it (6 patients [14.3%]), and would do it again (4 patients [9.52%]). There was no significant difference in STAI from screening to preoperative surveys between patients who underwent the AR experience and were neutral, enjoyed, or strongly enjoyed the experience compared with those who did not enjoy or strongly did not enjoy it (39 patients vs 5 patients; mean [SD] difference, −2.7 [7.3] vs −1.8 [8.4]; *P* = .71). Of patients who received the AR intervention, there were no adverse effects, such as visually induced motion sickness, experienced.

## Discussion

Our randomized clinical trial focused on the effect of AR on the patient’s entire experience at our institution’s outpatient surgical center, from initial outpatient preoperative visit to completion of the scheduled surgery, and its effects on perceived anxiety and satisfaction. Our custom AR experience allowed a patient to visually walk through their day at the surgery center with narration from their surgeon, and it was designed to emulate what the patient would encounter on the day of surgery. This experience included a visual representation of the building, layout of the surgical center, progression from check-in to preoperative care, to the operating room, and finally postoperative care. Furthermore, a strength of our AR technology was that patients had the opportunity to revisit modules of the AR experience if they wanted to view them again. Users could also interact with the AR application by zooming in and out and rotating the model for a more personalized experience. The ultimate goal of our AR experience was to educate and prepare the patient for what to expect, potentially mitigating the cognitive and physiological burden of anxiety they perceived surrounding their surgery.

We found that our AR experience significantly decreased patients’ preoperative anxiety even immediately from the time of screening to after intervention completion, with lasting effects until the time of surgery. This is contrasted with the control group, who had little significant change in their screening to postintervention surveys but did experience an increase in anxiety from screening to preoperative surveys. These changes subsided after surgery, with all patients showing a decrease in anxiety from their screening survey, with no significant difference between the groups. The focus of our AR application was on the day-of-surgery events and the immediate preoperative experience, which might explain the lack of observed postoperative differences. However, this study was underpowered, so this lack of statistical power increases the risk of type II error, potentially masking a significant effect.

We also examined the AR experience’s effect on postoperative pain and narcotic use. There were no significant differences between groups on VAS pain scores or narcotic use. These results are limited by the fact that they were patient-reported at the time of their postoperative survey and could thus be influenced by recall bias. Our study could have been strengthened by contacting patients on postoperative day 1 to determine VAS and narcotic use at that time point. We conducted a post-hoc power analysis to assess whether this study was appropriately powered to detect a clinically meaningful difference in VAS, and we did not reach appropriate confidence for definitive conclusion for this comparison. Previous studies have suggested that pain scores might be improved with the assistance of VR.^20,21,22,23,25,26,35^ While our study showed no significant difference in pain or narcotic use, the required number of participants was not met, so a type II error may be present.

While most patients who completed the AR experience did enjoy it, some did not. Indeed, approximately 10% to 15% of patients disagreed or strongly disagreed that they enjoyed the experience, would recommend it, and would do it again. Specific factors cited for their criticism were disorientation associated with using the AR headset, as well as the quality of the animations present. Patients with glasses sometimes had difficulty with the headset and fitting their glasses inside it so that they could see it project across the room. Although visually induced motion sickness is a well-described phenomenon with VR and AR, no patients in our study experienced any.^36,37,38,39^

We additionally examined whether patient experience with AR had any association with their anxiety levels. Interestingly, not enjoying the AR experience was not associated with the intervention’s efficacy of decreasing preoperative anxiety, as there was no significant difference in STAI score from screening to the preoperative survey between patients who underwent the AR experience and were neutral, enjoyed, or strongly enjoyed the experience compared with those who did not enjoy or strongly did not enjoy it. This suggests that the mental exercise of experiencing the operative day walkthrough in AR has an anxiolytic effect and is not dependent on ultimate enjoyment.

To our knowledge, limited randomized clinical trials have investigated the utility of an AR educational experience to prepare a patient for their day of surgery. A 2019 study by Eijlers et al^35^ performed a single-blind randomized clinical trial to investigate whether VR as a preparation tool for elective outpatient surgery in children was associated with lower levels of anxiety, pain, and emergence delirium from anesthesia compared with a control group receiving traditional care without VR.^35^ On the day of surgery, children receiving VR were exposed to a realistic, child-friendly immersive virtual version of the operating room so that they could get accustomed to the environment and anesthesia procedures. No differences between groups were found in self-reported anxiety, pain, emergence delirium, or parental anxiety, suggesting that VR in the pediatric population is not able to overcome the fear and anxiety experienced by a child before surgery. One possible explanation for this could be that a child’s brain has not fully developed the higher-level processing capabilities needed to fully integrate a VR experience into their reality or the necessary coping skills to navigate the experience of being a surgical patient. Of note, all patients in the study by Eijlers et al^35^ were aged 4 to 12 years, all of whom were younger than our youngest patient (age 14 years).

Other studies have looked at VR and AR and patient education focusing on digital imaging and 3-dimensional models to educate the patient on the specifics of their involved anatomy and provide a detailed visual representation of the steps of the surgery, yet these were primarily interested in patient understanding of the disease process and not on the effect on perioperative anxiety.^40^ Thus, to our knowledge, our study is the first of its kind to investigate AR’s application to the patient’s perioperative experience and demonstrate that AR decreased perioperative anxiety.

### Future Directions

Future directions include the inclusion of multiple centers with multiple surgeons and expansion to other languages. Our current experience focused exclusively on the day-of-surgery experience and did not provide any postoperative instructions, such as wound care or physical therapy, so adding these components might also impact patient experience and potentially even outcomes.

### Limitations

Our study has some limitations. First, this study was performed at a single institution with a single surgeon, and it was custom built to depict the exact process our patients experience. Generalizability is thus limited, since patients at other centers or with other surgeons may not experience a benefit. Additionally, we did not control for the presence of previous surgical procedures between the groups: a patient who has undergone surgery previously might not experience as much anxiety as someone who has never undergone surgery and is not as engaged in the experience. Furthermore, since participation was voluntary, our results may have been influenced by self-selection bias. Once enrolled, some patients no longer wished to complete the surveys, and this accounted for a small but noticeable attrition rate between patients who completed the preoperative surveys and those who did not complete either the entire or portions of the postoperative survey. Additionally, the study was underpowered in both groups, which increases the risk of type II error and reduces our confidence in the findings that were not statistically significant.

## Conclusions

In this randomized clinical trial, the administration of a preoperative AR experience decreased preoperative patient anxiety, and with most patient enjoying the experience, but there was no significant effect on postoperative anxiety, pain levels, or narcotic use. These findings suggest that the use of AR may serve as a means of decreasing preoperative patient anxiety.

## Notes

Supplement 1.
Trial Protocol and Statistical Analysis Plan

Supplement 2. **eTable.** Patient-Reported VAS and Narcotic Pill Number at Postoperative Day 1 (POD1) and at Their Postoperative Visit

Supplement 3.
Data Sharing Statement
