# Supplementary material for: Augmented Reality for Perioperative Anxiety in Patients Undergoing Surgery: A Randomized Clinical Trial
Source: JAMA Netw Open. 2023 Aug 17;6(8):e2329310. doi: 10.1001/jamanetworkopen.2023.29310 (PMC10436133; doi:10.1001/jamanetworkopen.2023.29310)
Supplement: Supplement 1. — Trial Protocol and Statistical Analysis Plan [file jamanetwopen-e2329310-s001.pdf]

Patient Reported Outcomes, Satisfaction, and Understanding following Orthopaedic Ambulatory Procedures using Augmented/Virtual Reality as an Education Tool

1) Objectives/ Aims

- To ascertain patient-reported outcomes, satisfaction, and understanding surrounding Orthopaedic procedures using augmented reality (AR) or virtual reality (VR) as an educational tool
- To determine whether AR/VR educational tools for patient experience are superior to more traditional handouts and in-office or over-the-phone conversations for perioperative experience
- To ascertain whether instructions detailing perioperative experience using the different information avenues ease perioperative flow and minimize patient stress regarding their procedure.

Study Endpoints

1. Anxiety
    - **This is the primary endpoint of the study**
    - Description: As measured by the validated State-trait anxiety inventory (STAI) questionnaire which includes 20 questions scored 1-4 (almost never, sometimes, often, and almost always) and weighted according to question content. Scores range from 20 to 80 with higher scores corresponding to greater levels of anxiety.
    - Timeframe: This metric will be assessed at the preoperative appointment before and after the intervention as well as at the postoperative appointment at the 7-14 day mark following surgery.
2. AR perioperative experience
    - Description: As measured by the study questionnaire which ranges 1 – 5 with higher scores indicating higher levels of agreement based on the statements:
        - "I enjoyed the AR experience"
        - "I would recommend the AR experience"
        - "I would use the AR experience again"
    - Timeframe: This metric will be assessed at the postoperative appointment at the 7-14 day mark following surgery.
3. Post-operative pain
    - Description: As measured using a visual analog scale (VAS) and number of narcotic pills taken

- Timeframe: This metric will be assessed at the postoperative appointment at the 7-14 day mark following surgery as self-reported by the patient on post-operative day 1 and at the time of the post-operative appointment.

2) Study Background

Augmented reality (AR) and virtual reality (VR) have recently gained much interest in the public sector with applications in video gaming, sports, home layout, navigation, and many others.[1] AR has been defined as "the concept of digitally superimposing virtual objects onto physical objects in real space so individuals can interact with both at the same time".[2] VR, alternatively, substitutes a user's complete visual environment with a computer-generated (CG) landscape. Both AR and VR have recently had increasing applications to medicine including medical training,[3] psychology,[4] physical medicine and rehabilitation,[5] and surgical specialties such as neurosurgery and orthopaedic surgery.[6,7] These surgical applications rely largely on supplementing a surgeon's visual field to assist in surgical technique or hardware placement, but little work has been done with applying AR/VR systems to patient experience and satisfaction. To our knowledge, no studies have been performed that examine AR or VR's ability to impact patient education and experience.

Prior studies have shown that increased education and visual aids such as handouts and videos can serve to decrease perioperative patient anxiety.[8] We hypothesize that by applying the modern technology of AR and VR to perioperative patient education and experience, we can further decrease perioperative patient anxiety and improve patient satisfaction.

3) Inclusion and Exclusion Criteria

Inclusion Criteria:

All patients 12 years old or greater who have a scheduled ambulatory procedure with our IRB-approved Orthopaedic providers.

Exclusion Criteria:

Any patient not scheduled for procedures, or patients who belong to vulnerable populations such as minors, the cognitively incapacitated, and/or prisoners.

Have any condition that, in the opinion of the investigator, would compromise the well-being of the patient or the study or prevent the patient from meeting or performing study requirements.

4) Recruitment Procedures

An a priori power analysis was conducted with a previously published effect size of 0.56 to determine 52 patients needed in each group (104 total, alpha = 0.05, power = 0.80). We planned to recruit 60 patients in each group to offset the possibility of dropout or loss of follow-up.

Patients who are scheduled to have an Orthopaedic procedure with one of our IRB-approved providers will be identified on the day of their appointment. Patients at random will either be provided standard written procedure instructions and expectations for their procedure prior to receiving AR/VR training.

At the conclusion of the visit with the provider, the enroller of the study will ask the patient a series of questions regarding their experience with the appointment and overall anxiety surrounding their procedure in private. The patient will then undergo the appropriate education based on their randomized group (i.e. either standard education via existing preoperative materials or AR-based education) and be administered the same set of questions. Following the procedure at the first postoperative appointment, the enroller of the study will administer a similar set of questions at the first follow-up visit.

☒ A pre-screening process will be used to determine whether potential participants are eligible to participate.

An IRB-approved researcher will access the provider’s appointment schedule prior to the encounter to confirm if the patient is a minor or not. The further determination as to whether the patient meets our inclusion/exclusion criteria will be made during the verbal consent process. If a patient meets our exclusion criteria, they will not be enrolled in the study. Medical charts will not be necessary to screen for patients. For minors age 12-17, verbal consent will be obtained from the parents or legal guardian accompanying the child to the appointment. Following parental consent, assent will be obtained from the child: the study will then be described to the child in a manner suitable for the child’s age and ability to comprehend. All questions the child may have will be answered appropriately, and if the child does not wish to participate, they may not.

Study Design

Study Type – Interventional

Primary Purpose – Supportive care

Interventional Study Model – Parallel

Number of Arms – 2

Randomized or not: Randomized

Open-label or if blinded: Open-label

Number of participants – 120

Arms and Interventions

1. **Standard of care preoperative teaching and handouts (n = 60)**
   a. **Intervention: patients in this arm will receive the standard set of preoperative instructions and handouts.**

   b. **Time of intervention: This intervention will be administered at the preoperative appointment.**

2. **Augmented reality perioperative experiences (n = 60)**
   a. **Intervention: patients in this arm will receive the standard set of preoperative instructions and handouts in addition to experiencing the Augmented Reality educational tool's instructions.**
   b. **Time of intervention: This intervention will be administered at the preoperative appointment.**

5) Check the procedures that you will use to collect data *(include estimated time for each participant to complete all study activities)***.**

☒ **Surveys – Attach all surveys, including Demographic Forms, in Other Attachments Section.**

Once patients agree to partake in the survey, they become subjects in our study. An IRB-approved researcher will ask questions verbally and/or administer an electronic survey and note the answers of the subject on an IRB-approved data collection sheet. The entire interaction will last an estimated 10 minutes. Once the first and second surveys are complete, the subject's involvement in the study ends. There will be no follow-up.

6) Compensation

☒ Participants will not be compensated.

☐ Participants will be compensated

7) Consent Process

The consent process will be initiated with study participants prior to starting any research procedures. Participants will be given ample time to consider their agreement. The study team will be available to answer any question in the method the participant prefers to communicate. No one in a perceived coercive position in relation to the participant will engage in the consenting process. Consent will be obtained voluntarily prior to initiating any study procedures:

☒ Verbally – Describe the Consent Process: At the conclusion of their preoperative appointment, an IRB approved researcher will join the provider and the patient and introduce themselves. The researcher will then describe the background and purpose of the survey as well as the patient's rights as a participant. Finally, the patient will be asked if they wish to participate. If they agree, the patient becomes a subject in the study. For minors age 12-17, verbal consent will be obtained from the parents or legal guardian accompanying the child to the appointment. Following parental consent, assent will be obtained from the child: the study will then be described to the child in a manner suitable for the child's age and ability to

comprehend. All questions the child may have will be answered appropriately, and if the child does not wish to participate, they may not.

8) Risks to Subjects

Minimal, if any, risks are expected for study procedures given:

☒ Identity of the human subjects cannot readily be ascertained, directly or through identifiers linked to the subjects and/ or

☒ The topic addressed would not reasonably place the subjects at risk of criminal or civil liability or be damaging to the subjects' financial standing, employability, educational advancement, or reputation.

The information provided by the patient will not in any way be tied to any identifiers nor will individual responses be shared with providers. In addition, we believe the topic at hand, which is only patient preference regarding the use of Telehealth/appearance of providers on Telehealth, is of minimal risk and does not introduce any liability to the patient.

Adverse Events and Serious Adverse Events

Adverse events will not be collected.

9) Potential Benefits to Subjects

The participants who complete the preoperative training with the video or AR/VR may receive the benefit of a greater understanding of the perioperative process and possibly decreased anxiety.

10) Data Management and Confidentiality

☒ Individuals' responses/statements will not be linked to their identity. (No identifying information will be included on the documents/recordings and the documents/recordings will not be coded and linked to the individual's identity.)

☒ All identifiable electronic data will be maintained on an encrypted device requiring a password for access. Passwords will not be shared and will be protected from access.

Data Storage

Will data be stored for future use ***for other than research*** described in this protocol?
☒ Yes ☐ No

If yes, will the data that are stored be identifiable?

☐ Yes, the data will be identifiable

☒ No, the data will be completely anonymous.

☐ No, the data will be stripped of identifiers and will be coded. A separate link from data to identifiers will be maintained, but the link to the individual's identity will not be made available to those requesting data from the data bank and will be maintained separately from the data bank.

Where will the data be stored?

All data will be stored on a UM box account by an IRB-approved researcher on this protocol.

How long will the data be stored?

Data will be stored upon conclusion of the study/publication of the data, after which it will be deleted.

Who will have access to the data?

Only IRB-approved researchers in this study will have access to the data.

Describe the procedures to release data, including: the process to request a release, approvals required for release and who can obtain data.

The final dataset will not be released to subjects. Subjects may request a tabulation of their individual answers at the conclusion of the survey but there will be no contact with the subject after the conclusion of their visit.

NOTE: If you will be collecting data that are sensitive, you must use good **data security practices** to collect, store, and transport your data. Keep in mind that officials in other countries, as well as U.S. Customs and Border Protection, could potentially try to access data stored on a phone, laptop, or other device.

References

1. What Is Augmented Reality? The Franklin Institute. Published September 21, 2017. Accessed November 28, 2020. https://www.fi.edu/what-is-augmented-reality

2. Azuma RT. A Survey of Augmented Reality. *Presence: Teleoperators and Virtual Environments*. 1997;6(4):355-385. doi:10.1162/pres.1997.6.4.355

3. Chaballout B, Molloy M, Vaughn J, Brisson Iii R, Shaw R. Feasibility of Augmented Reality in Clinical Simulations: Using Google Glass With Manikins. *JMIR Med Educ*. 2016;2(1):e2. doi:10.2196/mededu.5159

4. Chicchi Giglioli IA, Pallavicini F, Pedroli E, Serino S, Riva G. Augmented Reality: A Brand New Challenge for the Assessment and Treatment of Psychological Disorders. *Comput Math Methods Med*. 2015;2015. doi:10.1155/2015/862942

5. Dunn J, Yeo E, Moghaddampour P, Chau B, Humbert S. Virtual and augmented reality in the treatment of phantom limb pain: A literature review. *NeuroRehabilitation*. 2017;40(4):595-601. doi:10.3233/NRE-171447

6. Chytas D, Malahias M-A, Nikolaou VS. Augmented Reality in Orthopedics: Current State and Future Directions. *Front Surg*. 2019;6:38. doi:10.3389/fsurg.2019.00038

7. Meola A, Cutolo F, Carbone M, Cagnazzo F, Ferrari M, Ferrari V. Augmented reality in neurosurgery: a systematic review. *Neurosurg Rev*. 2017;40(4):537-548. doi:10.1007/s10143-016-0732-9

8. The effect of anesthetic patient education on preoperative patient anxiety. *Regional Anesthesia and Pain Medicine*. 1999;24(2):158-164. doi:10.1016/S1098-7339(99)90078-0

Statistical Methods

1. Introduction

   Augmented reality (AR) and virtual reality (VR) have recently gained much interest in the public sector with applications in video gaming, sports, home layout, navigation, and many others.[1] AR has been defined as "the concept of digitally superimposing virtual objects onto physical objects in real space so individuals can interact with both at the same time".[2] VR, alternatively, substitutes a user's complete visual environment with a computer-generated (CG) landscape. Both AR and VR have recently had increasing applications in medicine including medical training,[3] psychology,[4] physical medicine and rehabilitation,[5] and surgical specialties such as neurosurgery and orthopaedic surgery.[6,7] These surgical applications rely largely on supplementing a surgeon's visual field to assist in surgical technique or hardware placement, but little work has been done with applying AR/VR systems to patient experience and satisfaction. To our knowledge, no studies have been performed that examine AR or VR's ability to impact patient education and experience.

   Prior studies have shown that increased education and visual aids such as handouts and videos can serve to decrease perioperative patient anxiety.[8] We hypothesize that by applying the modern technology of AR and VR to perioperative patient education and experience, we can further decrease perioperative patient anxiety and improve patient satisfaction.

2. Study Methods

   a. Study Design: Randomized Controlled Trial

   b. Study Arms

      Standard of care preoperative teaching and handouts (n = 60)

      - Intervention: patients in this arm will receive the standard set of preoperative instructions and handouts.
      - Time of intervention: This intervention will be administered at the preoperative appointment.

      Augmented reality perioperative experiences (n = 60)

      - Intervention: patients in this arm will receive the standard set of preoperative instructions and handouts in addition to experiencing the Augmented Reality educational tool's instructions.
      - Time of intervention: This intervention will be administered at the preoperative appointment.

   c. Blinding: None

   d. Randomization Process:

      Prior to study commencement, a randomization table was generated using Python 3.8.10 for a total of 200 participants by sequentially randomly drawing either 0 or 1 to denote the different study groups.

      Patients indicated for the study were randomized into one of the two arms by using sequential entries into the randomization table. Randomization was

performed by the person enrolling the patient (Rizzo, Costello, or Luxenburg).

e. Inclusion and Exclusion Criteria

Inclusion Criteria:

i. All patients 12 years old or greater who have a scheduled ambulatory procedure with our IRB-approved Orthopaedic providers.

Exclusion Criteria:

- Any patient not scheduled for procedures, or patients who belong to vulnerable populations such as minors, the cognitively incapacitated, and/or prisoners.
- Have any condition that, in the opinion of the investigator, would compromise the well-being of the patient or the study or prevent the patient from meeting or performing study requirements.

3. Statistical analysis

a. Sample Size and Power Calculation

An *a priori* power analysis was conducted to determine the number of patients needed to detect a difference in State-Trait Anxiety Inventory (STAI) scores between the groups. To detect a clinically meaningful difference of a change of 5 points in the STAI with a power of 0.8, a significance level of 0.05, and standard deviation of 8.9, 51 patients were needed in each group. The standard deviation used was based on a separate study involving patients undergoing procedures

under general anesthesia.[9] With an expected attrition rate of 10-20%, we planned to enroll 60 patients in each group.

b. Study Endpoints

1. Anxiety – Primary Endpoint
   - Description: As measured by the validated STAI questionnaire which includes 20 questions scored 1-4 (almost never, sometimes, often, and almost always) and weighted according to question content. Scores range from 20 to 80 with higher scores corresponding to greater levels of anxiety.
   - Timeframe: This metric will be assessed at the preoperative appointment before and after the intervention as well as at the postoperative appointment at the 7-14 day mark following surgery.
2. AR perioperative experience – Secondary Endpoint
   - Description: As measured by the study questionnaire which ranges 1 – 5 with higher scores indicating higher levels of agreement based on the statements:

     "I enjoyed the AR experience"

     "I would recommend the AR experience"

     "I would use the AR experience again"
   - Timeframe: This metric will be assessed at the postoperative appointment at the 7-14 day mark following surgery.
3. Post-operative pain – Secondary Endpoint

- Description: As measured using a visual analog scale (VAS) and the number of narcotic pills taken
- Timeframe: This metric will be assessed at the postoperative appointment at the 7-14 day mark following surgery as self-reported by the patient on post-operative day 1 and at the time of the post-operative appointment.

c. Analysis Plan

We aim to perform hypothesis testing to test the null hypothesis that there is no difference between the study arms with respect to each of the endpoints including change in STAI score between the different time points and VAS and number of narcotic pills taken. We additionally will compare the difference in demographic factors between the groups including age, sex, anatomic location, smoking history, and psychiatric comorbidity.

Continuous variables will be tested using the Mann-Whitney U, and categorical variables will be tested using the Fisher Exact test. In the case of the comparison of multiple continuous variables, analysis of variance (ANOVA) testing will be used. Significance will be assessed at the $p < 0.05$ level, and all p values will be 2-tailed.

d. Statistical Software

All data analysis will be performed using Python 3.8.10 and R 4.2.1. We plan to use the libraries RPy2 3.5.4, NumPy 1.23.3, and SciPy 1.9.1 for Python and Stats 4.2.1 for R. Figures will be generated using Microsoft Word and Python using

Matplotlib 3.6.0.

4. Data management

All survey data will be collected and managed using REDCap electronic data capture tools hosted at the University of Miami. Each patient survey contained the STAI in addition to additional questions for experience and pain for the final survey as appropriate.

Patient demographic information and enrollment details were stored on a HIPAA-compliant cloud file storage system (box.miami.edu) licensed by the University of Miami. Only study organizers had access to REDCap and the cloud file system.

References

1. What Is Augmented Reality? The Franklin Institute. Published September 21, 2017. Accessed November 28, 2020. https://www.fi.edu/what-is-augmented-reality

2. Azuma RT. A Survey of Augmented Reality. *Presence: Teleoperators and Virtual Environments*. 1997;6(4):355-385. doi:10.1162/pres.1997.6.4.355

3. Chaballout B, Molloy M, Vaughn J, Brisson Iii R, Shaw R. Feasibility of Augmented Reality in Clinical Simulations: Using Google Glass With Manikins. *JMIR Med Educ*. 2016;2(1):e2. doi:10.2196/mededu.5159

4. Chicchi Giglioli IA, Pallavicini F, Pedroli E, Serino S, Riva G. Augmented Reality: A Brand New Challenge for the Assessment and Treatment of Psychological Disorders. *Comput Math Methods Med*. 2015;2015. doi:10.1155/2015/862942

5. Dunn J, Yeo E, Moghaddampour P, Chau B, Humbert S. Virtual and augmented reality in the treatment of phantom limb pain: A literature review. *NeuroRehabilitation*. 2017;40(4):595-601. doi:10.3233/NRE-171447

6. Chytas D, Malahias M-A, Nikolaou VS. Augmented Reality in Orthopedics: Current State and Future Directions. *Front Surg*. 2019;6:38. doi:10.3389/fsurg.2019.00038

7. Meola A, Cutolo F, Carbone M, Cagnazzo F, Ferrari M, Ferrari V. Augmented reality in neurosurgery: a systematic review. *Neurosurg Rev*. 2017;40(4):537-548. doi:10.1007/s10143-016-0732-9

8. The effect of anesthetic patient education on preoperative patient anxiety. *Regional Anesthesia and Pain Medicine*. 1999;24(2):158-164. doi:10.1016/S1098-7339(99)90078-0

9. Bondy LR, Sims N, Schroeder DR, Offord KP, Narr BJ. The effect of anesthetic patient education on preoperative patient anxiety. Reg Anesth Pain Med. 1999;24(2):158-164. doi:10.1016/s1098-7339(99)90078-0
