# Supplementary material for: Augmented Reality for Perioperative Anxiety in Patients Undergoing Surgery: A Randomized Clinical Trial
Source: JAMA Netw Open. 2023 Aug 17;6(8):e2329310. doi: 10.1001/jamanetworkopen.2023.29310 (PMC10436133; doi:10.1001/jamanetworkopen.2023.29310)
Supplement: Supplement 2. — eTable. Patient-Reported VAS and Narcotic Pill Number at Postoperative Day 1 (POD1) and at Their Postoperative Visit [file jamanetwopen-e2329310-s002.pdf]

Supplemental Online Content

Rizzo MG, Costello JP, Luxenburg D, Cohen JL, Alberti N, Kaplan LD. Augmented reality for perioperative anxiety in patients undergoing surgery: a randomized clinical trial. *JAMA Netw Open*. 2023;6(8):e2329310. doi:10.1001/jamanetworkopen.2023.29310

eTable. Patient-Reported VAS and Narcotic Pill Number at Postoperative Day 1 (POD1) and at Their Postoperative Visit

This supplemental material has been provided by the authors to give readers additional information about their work.

© 2023 Rizzo MG et al. *JAMA Network Open.*

eTable. Patient-Reported VAS and Narcotic Pill Number at Postoperative Day 1 (POD1) and at Their Postoperative Visit

	AR (n = 24)	Control (n = 24)	P Value
VAS POD1 (SD)	6.8 (2.8)	5.8 (3.4)	0.38
VAS Post-Op Visit (SD)	2.7 (2.0)	2.2 (2.1)	0.37
Pills POD1 (SD)	1.5 (1.2)	1.2 (1.2)	0.31
Pills Total (SD)	1.8 (1.3)	1.9 (1.3)	0.64

N.B. These data were collected at a single time-point at the time of their post-operative survey. Standard deviations (SD) are reported after means.

© 2023 Rizzo MG et al. *JAMA Network Open.*
