# Supplementary material for: Augmented Reality for Perioperative Anxiety in Patients Undergoing Surgery: A Randomized Clinical Trial
Source: JAMA Netw Open. 2023 Aug 17;6(8):e2329310. doi: 10.1001/jamanetworkopen.2023.29310 (PMC10436133; doi:10.1001/jamanetworkopen.2023.29310)
Supplement: Supplement 3. — Data Sharing Statement [file jamanetwopen-e2329310-s003.pdf]

Data Sharing Statement

Rizzo, Jr. Augmented Reality for Perioperative Anxiety in Patients Undergoing Surgery. *JAMA Netw Open*. Published August 17, 2023. doi:10.1001/jamanetworkopen.2023.29310

Data
Data available: No
